# Tonoplast Sucrose Trafficking Modulates Starch Utilization and Water Deficit Behavior in Poplar Leaves

**DOI:** 10.1093/pcp/pcac087

**Published:** 2022-06-21

**Authors:** Scott A Harding, Trevor T Tuma, Kavita Aulakh, Maria A Ortega, Dong Ci, Yongbin Ou, Chung-Jui Tsai

**Affiliations:** Warnell School of Forestry and Natural Resources, University of Georgia, Athens, GA 30602, USA; Department of Genetics, University of Georgia, Athens, GA 30602, USA; Department of Plant Biology, Athens, GA 30602, USA; Warnell School of Forestry and Natural Resources, University of Georgia, Athens, GA 30602, USA; Department of Plant Biology, Athens, GA 30602, USA; Warnell School of Forestry and Natural Resources, University of Georgia, Athens, GA 30602, USA; Department of Genetics, University of Georgia, Athens, GA 30602, USA; Warnell School of Forestry and Natural Resources, University of Georgia, Athens, GA 30602, USA; Department of Genetics, University of Georgia, Athens, GA 30602, USA; Department of Plant Biology, Athens, GA 30602, USA; Warnell School of Forestry and Natural Resources, University of Georgia, Athens, GA 30602, USA; Department of Bioscience and Biotechnology, Beijing Forestry University, Beijing 100083, China; Warnell School of Forestry and Natural Resources, University of Georgia, Athens, GA 30602, USA; Department of Biotechnology, School of Life Science and Engineering, Southwest University of Science and Technology, Mianyang 621010, Sichuan, China; Warnell School of Forestry and Natural Resources, University of Georgia, Athens, GA 30602, USA; Department of Genetics, University of Georgia, Athens, GA 30602, USA; Department of Plant Biology, Athens, GA 30602, USA

**Keywords:** Antioxidant, Condensed tannin, Drought, Flavonoid, Redox, Starch, Turgor

## Abstract

Leaf osmotic adjustment by the active accrual of compatible organic solutes (e.g. sucrose) contributes to drought tolerance throughout the plant kingdom. In *Populus tremula* x *alba*, *PtaSUT4* encodes a tonoplast sucrose–proton symporter, whose downregulation by chronic mild drought or transgenic manipulation is known to increase leaf sucrose and turgor. While this may constitute a single drought tolerance mechanism, we now report that other adjustments which can occur during a worsening water deficit are damped when *PtaSUT4* is constitutively downregulated. Specifically, we report that starch use and leaf relative water content (RWC) dynamics were compromised when plants with constitutively downregulated *PtaSUT4* were subjected to a water deficit. Leaf RWC decreased more in wild-type and vector control lines than in transgenic *PtaSUT4*-RNAi (RNA-interference) or CRISPR (clustered regularly interspersed short palindromic repeats) knockout (KO) lines. The control line RWC decrease was accompanied by increased *PtaSUT4* transcript levels and a mobilization of sucrose from the mesophyll-enriched leaf lamina into the midvein. The findings suggest that changes in *SUT4* expression can increase turgor or decrease RWC as different tolerance mechanisms to reduced water availability. Evidence is presented that PtaSUT4-mediated sucrose partitioning between the vacuole and the cytosol is important not only for overall sucrose abundance and turgor, but also for reactive oxygen species (ROS) and antioxidant dynamics. Interestingly, the reduced capacity for accelerated starch breakdown under worsening water-deficit conditions was correlated with reduced ROS in the RNAi and KO lines. A role for PtaSUT4 in the orchestration of ROS, antioxidant, starch utilization and RWC dynamics during water stress and its importance in trees especially, with their high hydraulic resistances, is considered.

## Introduction

Leaf osmotic adjustment (OA) can be defined as a decrease in bulk osmotic potential (Ψπ) due to increased solute accrual (Hsiao et al. 1976). With increasing water deficit, damaging turgor loss can be delayed or avoided depending on OA magnitude and on adjustments in cell wall elasticity (Kozlowski and Pallardy 2002). Both inorganic and organic solutes contribute to osmotic potential and adaptation to dry environments, but uptake of ionic (inorganic) species from the soil is not likely to increase during drought. Increased organic solute accrual in response to drought varies among *Populus* species, consistent with genetic influences (Barchet et al. 2014, Tschaplinski et al. 2019). Environmental factors including the speed of drought onset and its duration also influence OA in *Populus* (Gebre et al. 1994, Tschaplinski and Tuskan 1994, Barchet et al. 2014, Tschaplinski et al. 2019). Sugars, especially sucrose, are nearly always involved, with leaf soluble sugar contents increasing as much as 4-fold in drought-stressed compared to well-watered *Populus* in controlled environments (Tschaplinski and Blake 1989). Other compatible solutes such as proline and glycine-betaine often increase in response to drought, but not necessarily to osmotically impactful concentrations (Hare et al. 1998). Proline can increase to sub-millimolar concentrations, and glycine-betaine sometimes does not increase at all in abiotically stressed leaves of *Populus* (Guo et al. 2010, Ma et al. 2016). The utilization of starch for production of sugar solutes varies between *Populus* genotypes depending on their drought tolerance (Cao et al. 2014).

The true basis for OA is difficult to ascertain in water-stressed leaves because solute concentration increases can be due to various combinations of tissue shrinkage, distant sink demand and local metabolic activity (Turner 2018). Increased accrual of carbohydrate solutes by starch degradation or decreased growth relative to photosynthesis are common drivers of OA (Blum 2017). Another possibility is that OA can occur due to a change in intracellular sucrose trafficking. This idea is supported by findings that RNAi-knockdown (KD) or knockout (KO) mutants of *SUT4* encoding a tonoplast sucrose-effluxing transporter exhibit increased leaf sucrose contents in rice, poplar and maize (Eom et al. 2011, Payyavula et al. 2011, Leach et al. 2017). Moreover, a large sucrose increase along with a sharp decrease in *PtaSUT4* transcripts has been observed in leaves of wild-type (WT) poplar subjected to chronic mild drought (Frost et al. 2012). *PtaSUT4*-KD poplars also exhibit greater leaf water retention than WT leaves under increasing pressure (Harding et al. 2020). While there are multiple reports of *SUT4* downregulation in response to water stress (Xu et al. 2018), expression of *PtaSUT4* orthologs in sweet potato and *Arabidopsis* is positively regulated by abscisic acid (ABA) abundance (Gong et al. 2015, Wang et al. 2020). Furthermore, a direct connection between a tonoplast SUT and ABA-mediated starch utilization has been reported in apple (Ma et al. 2017). Increased *SUT4* expression has also been reported for xylem tissues of severely drought-stressed poplar (Pagliarani et al. 2019). Residual *PtaSUT4* expression increased in *SUT4*-KD poplars under a short term but acute drought stress, and ABA gene networks were perturbed (Xue et al. 2016). Leaf sucrose levels did not increase during the drought phase of the *SUT4*-KD poplar study, but a transient cytosolic OA would be possible since the vacuole can contain more than two-thirds of leaf mesophyll sucrose (Nadwodnik and Lohaus 2008, Fink et al. 2018). Overall, it appears that water deficits can lead to a spectrum of SUT4 responses. In trees with their high hydraulic resistances (Kozlowski and Pallardy 2002), SUT4 can be reasoned to have an important role in governing internal allocations of scarce water in accordance with sink and source requirements for plant-wide stress tolerance.

Such allocations would presumably be osmotically driven, but how SUT4 might participate in such osmotic dynamics has not previously been explored. Here we extend our earlier findings by reporting effects of *PtaSUT4* manipulation on sucrose abundance in petiole exudates versus bulk tissue. Further, we explore evidence of an interaction between sucrose accrual due to PtaSUT4, antioxidant abundance and starch metabolism. Finally, relative water content (RWC) changes during onset of water deficits were found to differ in relation to bulk osmotic changes between leaves of poplar plants with suppressed or natural expression of *PtaSUT4*. We discuss PtaSUT4 as an enabler of metabolic, turgor and osmotic shifts, which might buffer source leaf water demand relative to that of sinks in accordance with water deficit severity.

## Results

### Confirmation of CRISPR-induced *PtaSUT4-*KO lines

We previously reported on RNAi-KD lines with ∼25% residual *PtaSUT4* expression in *P. tremula* x *alba* INRA 717-1B4 (Payyavula et al. 2011, Xue et al. 2016). To increase the phenotype severity, we generated KO mutants in the same genetic background using CRISPR/Cas9 with a guide RNA targeting the first exon of *PtaSUT4* (**Supplementary Fig. S1A**) following established protocols (Zhou et al. 2015). Thirty independent transgenic lines were obtained, and amplicon deep sequencing confirmed biallelic mutations in all events (**Supplementary Fig. S1B**). Small insertions and deletions (indels) were the predominant mutations, especially 1-bp insertions (+1) and deletions (−1), consistent with published results (Bewg et al. 2018). The resulting transcripts with frameshift mutations and premature stop codons can be degraded by a quality control mechanism called nonsense-mediated messenger RNA decay (Popp and Maquat 2016, Tsai 2021). Indeed, low levels of ‘apparent’ *PtaSUT4* transcripts were detected in three randomly selected frameshift mutants by reverse transcription quantitative polymerase chain reaction (RT-qPCR) (**Supplementary Fig. S1C**). Residual transcripts harboring premature stop codons are not expected to produce functional proteins (**Supplementary Fig. S1D**). We thus conclude that all frameshift mutations are nonfunctional alleles. Hereafter and unless otherwise stated, all work was conducted using WT and Cas9-only control lines, an RNAi-KD line (KD) and two independent CRISPR-KO lines (KO) with frameshift alleles.

### Increased sucrose accumulation due to *PtaSUT4*-KD/KO was not limited to the vacuole

Downregulation of *PtaSUT4* by transgenic means or drought stress can cause sucrose levels to increase in poplar source leaves (Payyavula et al. 2011, Frost et al. 2012). How the increase is achieved or maintained is not clear. Vacuolar sequestration of sucrose as the sole basis for the leaf sucrose increase would cause a potentially disruptive osmotic imbalance within cells (Rennie and Turgeon 2009, Beauzamy et al. 2014). A simultaneous increase in cytosolic sucrose would work to alleviate such an osmotic imbalance, but would necessitate a means for limiting passive concentration-dependent symplasmic export (loss) of that sucrose to the sinks. To determine the effect of *PtaSUT4* manipulation on sucrose compartmentalization and tissue accrual, we examined tissue and petiole exudate abundance of source leaf sucrose and other osmolytes.

**Fig. 1:**
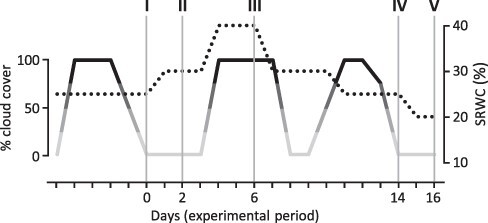
Schematic of treatment cycle and sampling dates. Changes in SRWC due to irrigation were monitored using soil moisture probes (dotted line). Changes in cloud cover are depicted as black (75–100%), dark gray (50–75%), medium gray (25–50%) and light gray (0–25%) traces. Roman numerals designate the five sample sets (dates) within the 16-day monitoring period. Sampling dates I, II and IV occurred on clear days following periods of overcast. Date V occurred when soil was at its driest and conditions had been sunny for several days. Date III occurred during an overcast period with frequent irrigation and saturated soil.

**Fig. 2:**
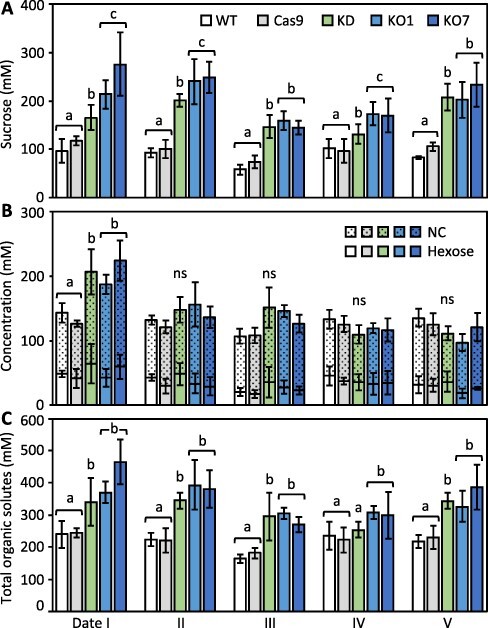
Effects of *PtaSUT4*-KD/KO on sucrose and overall solute concentrations in source leaves. **A**, Sucrose; **B**, hexose and abundant NC organic solutes; **C**, total organic solutes. Histogram bars represent mean ± SD of *n *= 4 plants per plant line at each sampling time point (date). Letters over histogram bar brackets indicate significant differences between WT-Cas9 pool (*n *= 8), KD pool (*n *= 4) and KO pool (*n *= 8) based on one-way ANOVA with post hoc multiple group comparison using Holm–Sidak with α = 0.05 and *P *< 0.05.

Three-month-old trees averaging 1.3 m in height were subjected to the sequence of cloud cover and soil moisture conditions depicted in **Fig. 1** (see Materials and Methods). Sucrose concentrations were higher in KD/KO lines at all dates, but date-to-date variation in bulk sucrose concentration followed similar trends in all lines (**Fig. 2A**). Sucrose concentration was ∼30% lower in all lines at date III (100% cloud cover) than at date II (0% cloud cover). Concentrations of other abundant metabolites, including the major hexoses fructose and glucose and various noncarbohydrates (NCs), referred to as [NC + hexose], generally differed much less between lines and dates, although relatively high in KD/KO at date I (**Fig. 2B**). The average estimated total organic solute concentration [OS] ranged from ∼220 mM to 320 mM to 350 mM for WT-Cas9 to KD to KO lines, with differences due largely to sucrose (**Fig. 2C**). Across all five dates, sucrose comprised ∼42.2% (93 mM) of the organic solute load in bulk molar terms in WT-Cas9 controls and ∼53.5% (170 mM) or 58.5% (206 mM) in KD or KO lines, respectively (**Fig. 2A**). By comparison, concentrations of the inorganic solute potassium averaged ∼70 ± 3.5 mM in leaves of all three plant groups and did not change under fluctuating soil moisture conditions of this experiment.

**Fig. 3:**
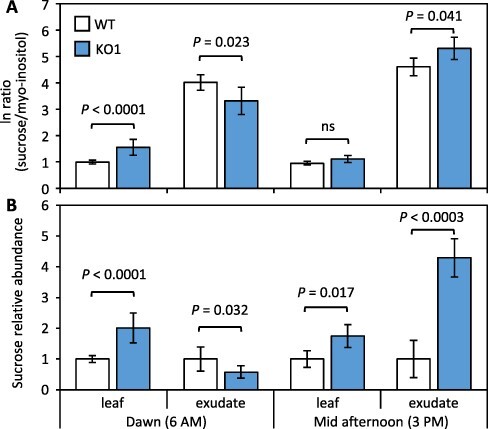
Source leaf tissue and petiole exudate sucrose analysis. **A**, Comparison of sucrose enrichment relative to myo-inositol in tissue (leaf) and petiole exudate (exudate) at dawn (6 AM) and mid-afternoon (3 PM). **B**, Leaf and exudate sucrose relative abundance. Histogram bars represent mean ± SE of *n *= 4 plants. The WT means were set to 1 to facilitate visual comparisons with proportionally adjusted KO data. *P*-values from two-sample *t*-test between genotypes are indicated. Exudate comparisons are also available from a second experiment with a KD for which corresponding tissue data were not collected (**Supplementary Fig. S2**).

In the event that increased sucrose sequestration within the vacuole accounted for the higher sucrose levels observed in KD/KO lines, a working hypothesis would be that the 30% bulk decrease occurring in all lines at date III was more at the expense of cytosolic sucrose, in proportional terms, in KD/KO than in WT-Cas9. Alternative scenarios are possible, but to further address the question of sucrose compartmentalization in the transgenics, sucrose abundance and sucrose:myo-inositol ratios in leaf tissue and petiole exudates were compared (**Fig. 3**). The approach of using exudates was taken because export phloem and mesophyll cytosol comprise a symplasmic continuum in so-called passive phloem-loading taxa like *Populus*, where sucrose abundance in transport phloem is driven by sucrose abundance in the leaf cytosol (Turgeon 2010, Carvalho et al. 2017). Myo-inositol was chosen for the ratio comparisons due to its substantial abundance relative to sucrose in gas chromatography-mass spectrometry (GC-MS) profiles of *Populus* leaves (Tschaplinski et al. 2019) coupled with its low abundance in phloem sap (Nelson et al. 1998, Amiard et al. 2004). Ratios of sucrose to myo-inositol were much higher in exudates than leaf tissues (**Fig. 3A**). Exudate sucrose enrichment was lower in KO than WT during the early morning, but increased several-fold during the day to become 2-fold higher in KO than WT exudates in the afternoon (**Fig. 3A**). Tissue abundance of sucrose was significantly higher in KO than WT at both time points but exudate sucrose abundance was highest in KO only in the afternoon (**Fig. 3B**). The ante meridiem (AM) data are consistent with the idea that *PtaSUT4*-KO facilitated vacuolar sucrose retention at the expense of exudate sucrose. We conclude that the reduction in AM exudate sucrose reflects a depletion of cytosolic sucrose at the end of the night and that the post meridiem (PM) exudate increase reflects increased cytosolic sucrose due to daytime photosynthesis. How *SUT4*-KO led to higher exudate sucrose than in WT later in the day raises questions that are discussed later. Greater sucrose enrichments relative to WT were also observed in PM petiole exudates collected from a KD line (**Supplementary Fig. S2**).

These conclusions partially rest on assumptions that contamination by vacuole contents and xylem sap were minimal and that the contributions of mass flow and diffusion to exudate collection were similar between lines. Caveats pertaining to the latter are discussed later. As to the former, exudate enrichment of sucrose relative to salicortin, a toxic phenolic glucoside abundant in leaves of Salicaceae species including *Populus* (Scriber et al. 1989), was used to assess vacuolar contamination. The peak area ratio (sucrose/salicortin) in whole tissue extracts from a random analysis of 18 petioles was ∼71 ± 12 (mean ± SE) whereas that in petiole exudates having detectable salicortin was ∼4,000 ± 390 (mean ± SE; *n *= 29). A separate comparison of free-flowing stem xylem sap with petiole sap collected using a pressure bomb device (see Materials and Methods) showed that sucrose was at least two orders of magnitude more abundant in the petiole than xylem sap (**Supplementary Fig. S3A**). Although sucrose contents in xylem sap were very low, they trended about 2-fold more abundant in KO compared to WT plants, and as a method control, 2-fold more abundant in intact versus 50% defoliated plants (**Supplementary Fig. S3B**). Myo-inositol was not detected in xylem sap. No salicortin was detected in petiole sap collected using the pressure bomb. These controls suggest little contamination of petiole exudate data by vacuolar sucrose or myo-inositol and little possibility of more than a negligible effect of petiole xylem sap on exudate sucrose determinations.

### Starch turnover was altered in KD/KO leaves

**Fig. 4:**
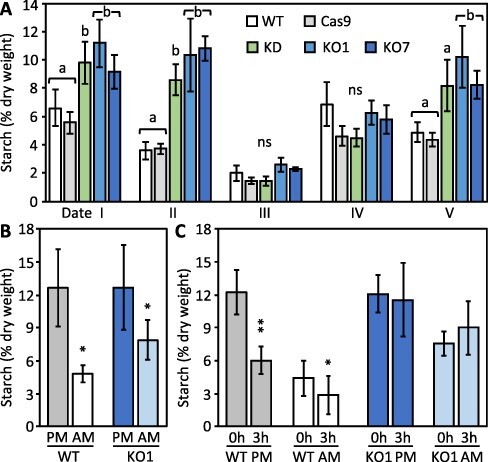
Starch homeostasis was perturbed in *PtaSUT4*-KD/KO source leaves. **A**, Starch; histogram bar definitions, pooling and significance testing as in **Fig. 2**. **B**, overnight starch turnover (net loss); **C**, starch turnover (net loss) during benchtop drying of excised leaves. Histogram data in B and C represent mean ± SD of *n* = 3 biological replicates. Statistical significance from the two-sample *t*-test between measurement times is indicated (*, *P* < 0.05; **, *P *< 0.01).

Previous work has shown little effect of *PtaSUT4*-KD on leaf midday starch levels under nonstress, hydroponic growth conditions (Payyavula et al. 2011). Based on those findings, altered sucrose compartmentalization dynamics do not necessarily perturb starch regulatory networks under conditions of zero water deficit. However, starch utilization may be important for drought tolerance in many species including poplars (Cao et al. 2014, Blum 2017). Therefore, leaf lamina starch levels were monitored with the objective of assessing *PtaSUT4* KD/KO effects on abundance under conditions of varying light and water availability (**Fig. 4A**). Starch levels tended to fluctuate similarly in all lines with changing cloud cover and soil moisture, but were most frequently higher in KD/KO than WT-Cas9 (**Fig. 4A**). Starch decreased to a minimum and did not differ significantly between lines under conditions of wetter soil and overcast weather at date III. With decreased cloud cover and increased soil drying after date III, lamina starch levels recovered in all lines. However, there was evidence of altered starch regulation in KD/KO lamina where abundance increased after date IV while remaining constant in WT-Cas9 (**Fig. 4A**). Midvein starch was substantially lower than lamina starch on a dry weight basis. In contrast to the dynamic situation in KD/KO leaf lamina, KD/KO midvein starch did not increase between dates IV and V (**Supplementary Fig. S4**)

We then assessed whether the pattern of higher starch levels in KD/KO leaf lamina reflected an altered sensitivity of starch metabolism to water availability. This was carried out in a separate experiment using a cohort of well-watered WT and KO plants. Leaf starch levels of the cohort plants were slightly higher than those of date III (water-replete) plants of the main monitoring experiment (**Fig. 4A**, **B**). PM starch levels of the lamina from the well-watered plants did not differ between WT and KO at the time of excision (**Fig. 4B**). AM starch levels trended slightly higher in KO, but this would not be attributed to water stress. Overnight starch decreases (between 7 PM and 7 AM) were significant in all lines but were nominally larger in WT than KO plants (**Fig. 4B**). In parallel, leaves from the same cohort were excised and subjected to a 3-h benchtop leaf drying procedure to determine whether water loss affected starch utilization differently between the lines. Starch levels declined sharply in WT but not the KO leaves during drying, regardless of whether leaves were excised at dawn or in the early evening (**Fig. 4C**). Excised WT and KO leaves remained pliant at the end of the drying cycle, having lost 14–16% of their dry weight during the process. The data are consistent with the idea that the elevated starch levels in KD/KO leaves reflected reduced starch degradation by some mechanism that is also sensitive to water deficit stress.

### *SUT4*-KD/KO altered antioxidant homeostasis

**Fig. 5:**
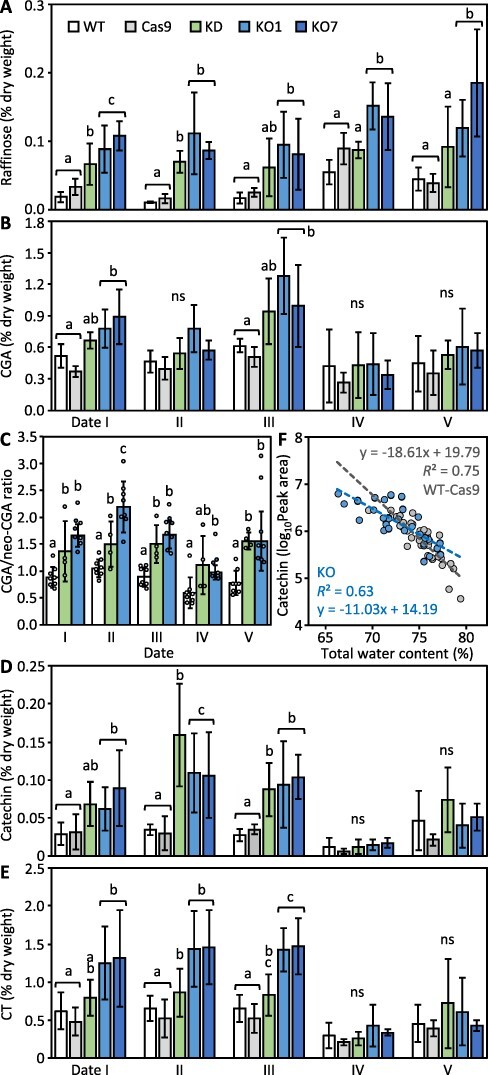
*PtaSUT4*-KD/KO increased antioxidant abundance and perturbed antioxidant pool composition. **A**, Raffinose; **B**, CGA; **C**, ratio of CGA/neo-CGA; **D**, catechin; **E**, CTs; **F**, catechin versus TWC regression. Catechin peak areas were log_10_-transformed to obtain normal data distribution. Date III data were excluded from the regression plots as described in Results. Histogram bar definitions, pooling and significance testing as in **Fig. 2**.

Starch homeostasis including its breakdown via amylases is sensitive to redox and reactive oxygen species (ROS) in both grain aleurone and leaf tissue (Scarpeci and Valle 2008, Suriyasak et al. 2017). Therefore, we compared leaf lamina antioxidant levels across the various lines (**Fig. 5**). Raffinose has antioxidant properties and, in addition to being a scavenger of harmful ROS species, is thought to be particularly well suited for the protection of chloroplast membranes (Nishizawa-Yokoi et al. 2008, Van den Ende 2013). Tissue abundance of raffinose was higher in KD/KO than WT-Cas9 at all sampling dates and also in leaves used for benchtop drying (**Fig. 5A** and **Supplementary Fig. S5**). Phenolic isomers chlorogenic acid (CGA, 5-*O*-caffeoylquinic acid) and neo-CGA (3-*O*-caffeoylquinic acid) are relatively abundant in poplar leaves (Tsai et al. 2006) and have similar antioxidative activities, but exhibit differences in their susceptibility to oxidation by diphenol oxidase (Radi et al. 1997, Nakatani et al. 2000). CGA and the ratio of CGA/neo-CGA were higher in KO, and to a lesser extent, in KD, than in the controls throughout the monitoring period (**Fig. 5B**, **C**).

Condensed tannins (CTs) comprise known ROS scavengers with additional roles in poplar defense and abiotic stress tolerance (Ullah et al. 2017, Gourlay and Constabel 2019, Harding 2019, Gourlay et al. 2022). Flavan-3-ol catechin along with the flavan-3-ol-derived CTs exhibited their greatest abundances in KO lines (**Fig. 5D**, **E**). Interestingly, catechin abundance correlated strikingly (*r^2^* = 0.63 and 0.75 for KD/KO and WT-Cas9; respectively) with leaf total water content (TWC) (**Fig. 5F**). The regression slope of catechin abundance versus TWC was nearly 2-fold greater (*t*-value = 3.08; *P *= 0.004) in WT-Cas9 than KD/KO plants (note, date III data clustered away from the respective regression lines for unclear reasons and were excluded). Catechin levels were also higher in KO than WT leaves used for the benchtop drying experiment (**Supplementary Fig. S5**). With our interest in whether antioxidant levels could affect starch degradation via ROS scavenging, the ROS species H_2_O_2_ was measured in the benchtop drying experiment and found to be substantially lower in KO than WT (**Supplementary Fig. S5**).

Midvein phenolic antioxidant levels were substantially lower than in lamina, 90% lower in the case of the most abundant antioxidant, CGA (**Supplementary Fig. S6**). Raffinose levels were not lower in midvein than lamina, but levels of the raffinose precursor galactinol were 75% lower than in lamina. Entry and retention of raffinose in transport phloem sieve elements are much greater than those for galactinol (Ayre et al. 2003). From this, it is concluded that most midvein raffinose was not synthesized there but was present in sieve elements of the transport phloem. Therefore, cytosolic antioxidant levels in the mesophyll were likely much lower in midvein than lamina. Overall, *PtaSUT4*-KD/KO led to elevated antioxidant levels primarily in leaf lamina where the starch increases relative to WT-Cas9 was observed.

### Hydration of leaves excised from water-stressed plants was impaired in *SUT4*-KD/KO

**Fig. 6:**
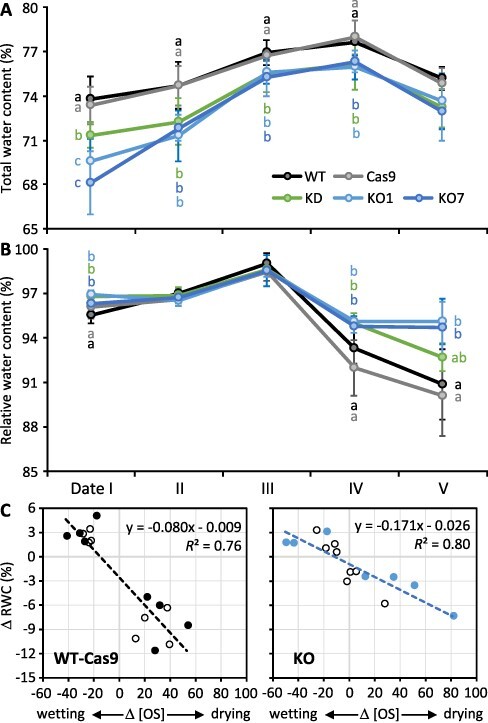
RWC was more sensitive to organic solute-level dynamics in WT-Cas9 than KD/KO leaves during drought onset. **A**, TWC; **B**, RWC; **C**, ΔRWC versus Δ[OS] for WT-Cas9 (left) or KO plants (right). Symbols in **A** and **B** represent mean ± SD of *n *= 4 plants of each line. Genotypes with the same letter at a given date were not significantly different from each other based on post hoc multiple group comparison using Holm–Sidak with α = 0.05 and *P *= 0.05. In **C**, positive ΔRWC values (*y*-axis) represent the RWC increase during wetting between dates I and III, while negative RWC values represent the decrease during drying between dates III and V. Negative Δ[OS] values (*x*-axis) represent the bulk organic solute concentration decrease as a percentage of initial [OS] during soil wetting between dates I and III. Positive values represent the increase during drying from dates III to V. Filled circles represent WT and KO7, and open circles represent Cas9 and KO1. The regression slopes differed significantly between WT-Cas9 (left) and KO (right) panels (*P *= 0.0014).

Ramifications of dynamic sucrose compartmentalization for starch and leaf water homeostasis and stress tolerance were further explored by monitoring RWC. RWC correlates with water potential, and it decreases when there is a concentrating effect of water loss on leaf solutes or when leaf solute concentrations increase without a corresponding increase in water. RWC was estimated by water uptake through the petiole (hydration) of an excised leaf (see Methods). An experiment was conducted to determine whether the leaf RWC response to changes in soil water content differed between KD/KO and WT-Cas9 leaves. For context, leaf TWC was lower in KD/KO than WT-Cas9 throughout the monitoring period, but oscillated similarly in all lines in response to changes in soil water content (**Fig. 6A**). As soils transitioned from very wet to dry (dates III–V), RWC decreased substantially more in WT-Cas9 than KO leaves, with KD falling in between (**Fig. 6B**), even though TWC tracked almost identically in all lines. The RWC decrease of date IV preceded the TWC decrease (date V).

To determine whether the larger RWC decrease of WT-Cas9 was due to a larger increase in organic solute concentration [OS], the RWC change (ΔRWC) was plotted versus the concurrent change in organic solute concentration (Δ[OS]). In this case, a 40% [OS] increase roughly equated to an RWC decrease of ∼7% in WT-Cas9 and a decrease of ∼3% in KO leaves (**Fig. 6C**). As a note, petioles of younger leaves exhibited daytime flaccidity at date V, but no source leaf wilting occurred during the course of monitoring. The larger [OS]-normalized RWC decrease in WT-Cas9 than KO during soil drying is consistent with there being a larger effect of a given [OS] increase on RWC in WT-Cas9 than KD/KO leaves. Although leaf water loss (ΔTWC) was equal in all lines between dates IV and V, replacement of lost water under RWC measurement conditions occurred more readily (RWC was lower) in WT-Cas9 than KD/KO leaves.

**Fig. 7:**
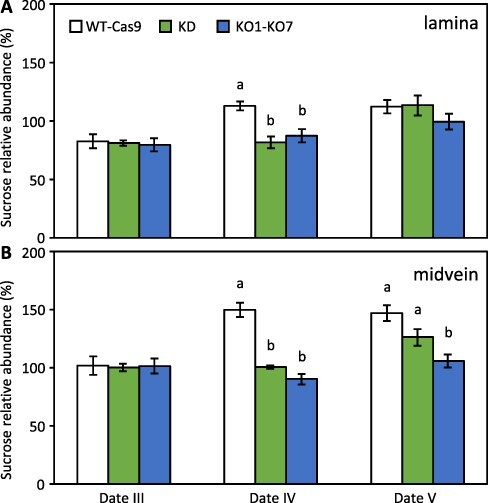
Spatial redistribution of leaf sucrose during drought onset. **A,** Sucrose abundance changes in deveined lamina tissue during soil wetting (date III) and drying (dates IV–V); **B,** sucrose abundance changes in midvein (as for lamina). Comparisons are on a dry weight basis using repeated measures across dates for each plant, with the sucrose content of each plant at date II set as 100%. Histogram bars represent mean ± SD. Letters indicate significant differences between WT-Cas9 pool (*n *= 8), KD pool (*n *= 4) and KO pool (*n *= 8) based on one-way ANOVA with post hoc multiple group comparison using Holm–Sidak with α = 0.05 and *P *< 0.05.

The RWC data led us to hypothesize that on dates IV and V WT-Cas9 leaves rehydrated more readily than KD/KO leaves at least partly because of PtaSUT4-mediated sucrose trafficking into the cytosol within individual cells. An additional possibility made more likely by a cytosolic sucrose increase is that redistribution of sucrose within the leaf during soil drying was greater in control than mutant leaves and contributed to the RWC decrease. Sucrose redistribution was examined by comparing lamina and midvein sucrose contents during the period of gradual soil drying and initial RWC decreases. On a dry weight basis, sucrose abundance increased slightly but transiently in the lamina portion of WT-Cas9, but not KD/KO leaves, at date IV (**Fig. 7A**). A larger sucrose increase was observed in the midvein at dates IV and V of WT-Cas9 and a smaller one at date V of KD. No response was observed for KO midvein (**Fig. 7B**). There was no evidence that photosynthesis increased between dates IV and V as a response to soil drying. In line with previous data that photosynthesis was affected similarly by mild drought in WT and KD plants (Frost et al. 2012), chlorophyll fluorescence (PSII quantum yield) did not differ significantly between KO and WT at dates IV or V (**Supplementary Fig. S7**). It is noted, however, that the photosystem II (PSII) quantum yield decrease between dates IV and V was statistically significant for KO (**Supplementary Fig. S7**).

### *PtaSUT4* transcript level dynamics and leaf RWC

**Fig. 8:**
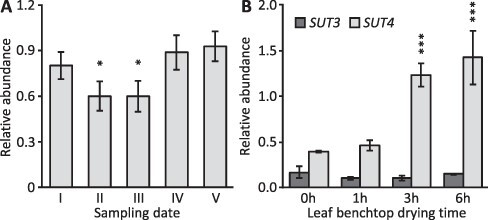
*PtaSUT4* expression dynamics in WT-Cas9 source leaves. **A**, *PtaSUT4* transcript levels during the monitoring period. Values represent mean ± SE of *n *= 6 plants, three WT and three Cas9 controls for each of the five sampling dates. Significant difference (*P* < 0.05) from the two-sample *t*-test was indicated above histogram bars for dates II and III (wetter soil) versus date V (dryer soil). **B**, *PtaSUT4* transcript levels during benchtop drying of excised leaves. Expression changes of *PtaSUT3* encoding a plasma-membrane SUT are also shown for reference. Data represent mean ± SD of *n *= 3 biological replicates. Significant difference (*P *< 0.001) from the two-sample *t-*test was indicated for 3 h and 6 h versus 0 h.

The data so far compare plants with normal versus constitutively altered *PtaSUT4* expression (KD/KO). Whether the above-described RWC responses by WT-Cas9 (**Figs. 6** and **7**) to a developing water deficit involved a dynamic *PtaSUT4* response was therefore examined. Transcript levels increased during the period of soil moisture decreases from dates III to IV–V (**Fig. 8A**) and increased sharply in parallel with a 14–16% fresh weight loss during benchtop drying of excised leaves (**Fig. 8B**). From these and the RWC data (**Fig. 6B**, **C**), we suggest that vacuole/cytosol partitioning of sucrose can be modulated by PtaSUT4 with consequences for longer-distance transport and leaf RWC.

## Discussion

### A possible basis for PtaSUT4-mediated OA

Our results were consistent with a promotive effect of *PtaSUT4* downregulation on vacuolar sucrose sequestration (AM response) coupled with an ability of KO to accrue a several-fold higher cytosolic sucrose concentration than WT (PM response) (**Figs. 2** and **3**). How this could happen in light of the symplasmic continuum between leaf mesophyll and export phloem is still a matter for speculation. We have previously reported increased cellular sphericity in the mesophyll of KD leaves along with a higher bulk modulus of elasticity compared to WT leaves (Harding et al. 2020). One possible outcome is greater turgor and reduced contact area between KD cells for intercellular plasmodesmatal connections and symplasmic flow, in accordance with principals reviewed in Schulz (2015). Consistent with the possibility of limited export, growth of *PtaSUT4*-KD plants was more reduced than that of WT plants when photosynthetic capacity was reduced by partial defoliation (Harding et al. 2020). How reduced intercellular contact would lead to comparatively greater solute exudation by KO than WT leaves after excision, but greater solute retention in attached leaves, perhaps at the expense of sink supply, is not clear. However, excision of a transpiring leaf results in hydrostatic failure and alters the pressure gradient between plasmodesmatally connected cells. Plasmodesmata are mechanosensitive and seal upon the sudden development of a pressure differential (Park et al. 2019). Whether hydrostatic tension was weaker in KO than WT leaves before excision, thus delaying or otherwise inhibiting phloem sealing in KO leaves after their excision, was not determined, but hydrostatic behaviors of *PtaSUT4*-KD and WT leaves have been found to differ (Harding et al. 2020).

### Antioxidants and starch regulation

Leaf starch levels were higher in KD/KO than WT-Cas9 except for the two dates of highest leaf TWC (III and IV) in the greenhouse monitoring experiment (**Figs. 4A** and **6A**). Data from the excised leaf drying experiment suggests that slower degradation in the dark, slowed further by water loss, was probably a contributing factor to higher KO starch levels (**Fig. 4C**). Starch synthesis and degradation are sensitive to cell redox (Skryhan et al. 2018), which on the basis of antioxidant and H_2_O_2_ levels may have been perturbed in lamina of KD/KO source leaves (**Fig. 5**; **Supplementary Figs. S5** and **S6**). ROS resulting from drought stress of leaves or during development (e.g. seed germination) has been shown to have direct promotive effects on β-amylase and starch degradation (Scarpeci and Valle 2008, Ishibashi et al. 2012, Prasch et al. 2015, Zanella et al. 2016). In vegetative tissues, raffinose produced in the cytosol from sucrose and galactinol is taken up by chloroplasts and known to have a role in ROS neutralization (Nishizawa-Yokoi et al. 2008, Schneider and Keller 2009, Findling et al. 2015). Besides ROS neutralization, there are additional potential mechanisms by which phenolic antioxidants may interfere with starch degradation. Catechin and its downstream metabolites occur in the chloroplast where starch granules form (Brillouet et al. 2013). Hydroxycinnamoyl-CoA quinate transferase isoforms for CGA biosynthesis have been localized on starch granules with CGA exhibiting α-amylase inhibition at low (half maximal inhibitory concentration or IC50) levels (Li et al. 2019, Zheng et al. 2020). Catechin and other flavan-3-ols inhibit α-amylase at sub-millimolar levels (20 μM) (Forester et al. 2012).

A confounding factor in our analysis is that raffinose and phenolic antioxidant levels are themselves promoted by sucrose abundance (Karner et al. 2004, Solfanelli et al. 2006, Van den Ende 2013). Sucrose also has a central role in trehalose-6-phosphate signaling with a possible influence over leaf starch degradation in the dark (Martins et al. 2013, Griffiths et al. 2016). Sucrose and SnF1 (sucrose non-fermenting1)-like kinase have long been connected with the redox regulation of starch metabolism (Geigenberger et al. 2005), with a number of steps in starch metabolism now known to be ABA and redox-sensitive (Thalmann et al. 2016, Skryhan et al. 2018). Potentially illuminating instances in which date-to-date changes in sucrose and antioxidant abundance were not coupled did occur in the present dataset. For example, date IV lamina raffinose increases were concurrent with phenolic antioxidant decreases (**Fig. 5**). This suggests the possibility that diversification of the antioxidant response can occur independently of sucrose and perhaps modulate certain redox effects of sucrose including those on starch use.

The close correlation between catechin and TWC (**Fig. 5F**) along with evidence for an inhibitory effect of antioxidants on starch breakdown adds to an unexplored interface between starch metabolism, phenolics and leaf water control. Flavonoids can promote stomatal opening via ROS scavenging (Watkins et al. 2017, Li et al. 2021) and have also been proposed to act as osmolytes in their own right, able to contribute to CT accrual in chloroplasts for both osmotic and ROS scavenging services (Chalker-Scott 2002, Brillouet et al. 2013, Harding 2019). The capacity of antioxidants to prevent source leaf injury, tune gas exchange, modulate starch breakdown and its osmotic amplification of source leaf water demand could shift protection between source and sink organs depending on their respective water requirements. For example, higher RWC and thus lowered water demand by source leaves due to reduced *PtaSUT4* expression under certain drought conditions would minimize hydraulic shocks to sink meristems. On the other hand, increased source leaf water demand facilitated by increased PtaSUT4 capacity under conditions severe enough to halt or reduce sink growth might be important for source involvement in the overall plant survival and post-stress recovery.

### A role for SUT4 in OA

**Fig. 9:**
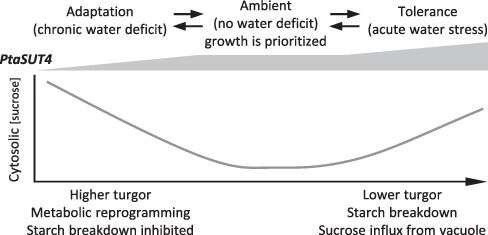
A proposed model. Varied *PtaSUT4* expression during water stress may support alternative tolerance strategies. *PtaSUT4* downregulation (left-hand side) by transgenic means can lead to increased sucrose sequestration in the cytosol and vacuole and increased turgor, modulus of elasticity, Ψ*p*, raffinose and phenolic antioxidants and starch. These features may all contribute to steady, albeit reduced, growth during persistent mild water deficit conditions. With more severe water deficit (right-hand side), increased *PtaSUT4* transcript levels and a strong contribution by starch degradation elevate cytosolic solute levels and promote sucrose movement from the mesophyll toward the vasculature, potentially aiding leaf ability to hydrate. This response may enable night-time recovery during a nonlethal drought and during recovery upon restored water availability. In unstressed WT plants prioritizing their growth (middle), ambient *PtaSUT4* expression limits sucrose sequestration by source leaves, which means that under normal transpiring conditions the export to sinks is more efficient than in the case of leaves with reduced *PtaSUT4* expression.

A scenario is offered in which SUT4 participates in poplar drought tolerance (**Fig. 9**). WT *PtaSUT4* expression under nonstress conditions (**Fig. 9**, center) enables efficient carbon export for when growth is being prioritized. Source leaf retention of carbohydrates and resource allocations for antioxidants are kept in line with what is necessary for turgor, protection and rapid growth. With drought onset, *PtaSUT4* expression can increase or decrease. A *PtaSUT4* increase facilitates in principle a cytosolic sucrose increase at the expense of vacuole sucrose. Under dryer conditions and increased starch degradation, starch-derived sucrose would partition away from the vacuole, act as a compatible solute in the cytosol and spread symplasmically from the mesophyll into the transport phloem, potentially facilitating hydration at night or during drought relaxation (**Fig. 9**, right). In the event that mild water-deficit conditions cause a natural reduction in leaf *PtaSUT4* expression (Frost et al. 2012), increased vacuolar sucrose and turgor may increase RWC and reduce source leaf competition with meristematic sinks for water (**Fig. 9**, left). Sucrose from starch use could contribute toward either an RWC decrease or an RWC increase depending on a combination of time-of-day, *PtaSUT4* expression and effect of that on antioxidant–ROS homeostasis. We speculate that SUT4 participation in osmolyte compartmentalization may be especially important in tree species where changes in localized osmotic gradients against a backdrop of large hydraulic resistances might be critical for governing water allocations between source and sink organs.

## Materials and Methods

### Production of CRISPR lines

*PtaSUT4*-KO lines of *P. tremula* x *alba* clone INRA 717-1B4 were generated by CRISPR/Cas9 according to the approach of Zhou et al. (2015) with a single gRNA sequence (GTCATTCCGCTGACATTGGG) under control of the *Medicago truncatula U6* promoter in p201N-Cas9 (Addgene 59175, gift of Wayne Parrott). *Agrobacterium tumefaciens*-mediated transformation of 717 was performed as described (Bewg et al. 2022). Mutation patterns were analyzed by amplicon sequencing following the protocol of Jacobs et al. (2015), using primers CCTACACGACGCTCTTCCGATCTACCAGCCGGTTTGGAAG and GTTCAGACGTGTGCTCTTCCGATCATTAGCAACATCGAGAATCCAA and sequenced on an Illumina MiSeq. The data were then processed by AGEseq (Xue and Tsai 2015) and biallelic mutations were confirmed in all cases (**Supplementary Figure S1**).

### Plant propagation and growth

Single-node cuttings were grown in a glasshouse in 1-gallon pots containing commercial soil mixture (Fafard 3B) supplemented with Osmocote (15-9-12 NPK 4 month release). For the experiments, four copies of each of five genotypes, WT, Cas9 vector control, an RNAi-KD line with 25–35% residual *PtaSUT4* expression used in previous work (Frost et al. 2012, Xue et al. 2016, Harding et al. 2020) and two KO lines were equally spaced but randomly positioned on a floor area of 6 m × 3 m. Plants were grown to ∼1.0–1.5 m in height with daily drip irrigation, supplemental light emitting diode (LED) lighting to maintain a 14-h photoperiod with a photon flux density in the upper canopy of ∼600 μmol photons m^−2^ s^−1^ on overcast days, but in mid-lower canopy, only 150 μmol on overcast days, with temperature control to prevent night-time temperatures from dropping below 18°C and to keep daytime temperatures above 30°C on overcast days. During the treatment phase of the experiment, outdoor daytime temperatures averaged 11.6 ± 4.2°C. Cloud cover varied as shown in **Fig. 1A**. Three days before leaf sampling commenced, leaf number was reduced to 30 fully expanded leaves per plant by removal of the required number of fully expanded leaves below the 20th internode (4–8 leaves). This was done in order to achieve a uniform canopy area and equalize pot water demand. Leaf harvesting commenced with the leaf just above the pruned zone and progressed up the growing stem. During the sampling phase of the experiment, there was a net loss of approximately one fully expanded leaf per plant. All plants were sampled in the same way at each of the five sampling dates.

### Pot water control, leaf water content and leaf water potential

For the week before initial (date I) sampling, pots were maintained well-drained at about 20–25% soil relative water content (SRWC) using automated irrigation with daily monitoring with soil tensiometers (Decagon EC-5) and manual watering as necessary. In previous work using 4 gallon pots, it was determined that maximum water holding capacity was about 45% SRWC and that turgor loss in the most vulnerable leaves (leaves just approaching full expansion) began to develop at SRWC of 5–8% (Frost et al. 2012). After sampling date I, irrigation was varied, raising and lowering nominal SRWC approximately as indicated (**Fig. 1**). Tensiometer monitoring and manual watering were continued. Reversible turgor loss in petioles of vulnerable leaves above the sampling zone affected about 25% of the plants at date V.

### Leaf sampling

Two adjacent mature source leaves were harvested each sampling date at ∼8–9 AM, ∼3–4 h after supplemental lights were turned on. One leaf was snap frozen in liquid nitrogen for metabolite and RNA analysis and the other was weighed for determination of leaf RWC. Weighed leaves were then hydrated 4 h in a dark humid chamber with petioles submerged in dH_2_O. Leaf fresh weights were obtained in the greenhouse using a portable scale and ranged between 3.5 and 4.5 g, with a weighing error of ±5 mg (≤0.15%). Hydrated and final dry weights were obtained with an analytical balance (weighing error of ±0.5 mg) to minimize error compounding. Leaf dry weights were determined after 24 h in a 55^o^C forced air oven. RWC was calculated as (FW − dry weight)/(hydrated weight − dry weight) in percentage and TWC as (FW − dry weight)/FW in percentage.

### Leaf drying experiment

Source leaves were excised from a cohort of well-watered WT and KO1 plants at actual dawn (7 AM) and late in the afternoon (7 PM). Three WT and three KO1 leaves were immediately snap frozen and later freeze-dried for processing. Three leaves of each genotype were inserted into Falcon tubes filled with deionized water and placed in a dark humid chamber. Three leaves of each genotype were subjected to benchtop drying under still conditions with photon flux density (PFD) < 5 μmol photons m^−2^ s^−1^. After 3 h, all leaves were snap frozen and freeze-dried. Starch was determined for the humid chamber control and benchtop-treated leaves. Catechin, raffinose and H_2_O_2_ contents were determined for all leaves.

### Quantitative RT-PCR

RNA was extracted from source leaves using the Plant RNA Reagent (Life Technologies, ThermoFisher Scientific, Carlsbad, CA, 92008, USA) in conjunction with the Direct-zol RNA MiniPrep Kit (Zymo Research, Irvine, CA, 92614, USA). Complementary DNA (cDNA) was synthesized using the High-capacity cDNA Reverse Transcription Kit (Life Technologies). qPCR was performed using the ABsolute Blue QPCR SYBR Green Mix with ROX (ThermoFisher Scientific, Carlsburg, CA, 92008, USA) using primers for *SUT4* (Potri.002G106900, ATCCTTGGGACTTGGACAAGGTGG and TGATCGACGAATACCCAAGATGGC), *SUT3* (Potri.019G085800, TGGTKTCTGTAGCARSTGGACCTT and ACTAACTGCGGCTGCAACA) and housekeeping controls *EF1B* (elongation factor 1-beta, Potri.001G224700, GACCTKGTATCAGTGGATTCCCTC and GAACAGAGGCACAAGATTACCAGG) and *ARP* (actin-related protein, Potri.017G057400, ACTGTGAGGAGATGCAGAAACGCA and GCTGTGTCACGGGCATTCAATGYT) for **Fig. 8** or *eIF4E* (eukaryotic translation initiation factor 4E, Potri.013G057000, TTTGGGAGGATCTGGTTCTTG and GGGAAGCTTCAAGTGCCTTT) for **Supplementary Fig. S1C**. Relative *PtaSUT4* transcript levels were determined by the 2^−ΔCt^ method as described previously (Rajinikanth et al. 2007).

### Metabolite Analysis

Leaves were lyophilized for 48 h (FreeZone 2.5, Labconco, Kansas City, MO, 64132, USA) and then ground through a 40-mesh sieve using a Wiley mill (Thomas Scientific, Candler, NC, 28715, USA). Aliquots of the coarse powder were further ball-milled in a Mini-BeadBeater (Biospec 3110Bx, Biospec, Bartlesville, OK, 74005, USA) at intensity setting 25 for two cycles. Ten milligrams of the lyophilized powder was suspended in a microtube with 500 µl methanol:chloroform (1:1, v/v) containing ribitol as internal standard and sonicated for 15 min in a sonic bath with pre-chilled water (4°C). Deionized water (200 μl) was then added to the tubes and samples vortexed and re-sonicated for 5 min. After centrifugation, 10 μl of the upper aqueous-methanol phase was evaporated to dryness (Centrivap, Labconco) in 200 μl glass microserts and derivitized for GC-MS as described previously (Jeong et al. 2004). Briefly, the dried extract was methoximated in 15 μl methoxyamine hydrochloride/pyridine solution (20 mg/ml; Sigma-Aldrich) for 30 min at 30°C and then silylated for 90 min at 60°C after adding 30 μl *N*-Methyl-*N*-(trimethylsilyl) trifluoroacetamide (Sigma-Aldrich, St. Louis, MO, 63178, USA). Incubations were carried out in a Vortemp 56 orbital shaker (Labnet, Edison, NJ, 08837, USA) at 600 rpm. Derivitized samples were injected (1 µl) in 25:1 split (sucrose and hexoses) and splitless modes at an inlet temperature of 250°C. Metabolites were resolved on a DB-5MS column (30 m length, 0.25 mm diameter, with DuraGuard pre-column) with a helium flow of 1 ml/min. GC (Agilent Technologies, Santa Clara, CA, 95051, USA) oven temperature at injection was 80°C. Following a 1-min hold at 80°C, temperature was ramped 20°C/min to 200°C and then 10°C/min to 320°C with a 6.5 min hold at 320°C. Metabolites were detected using an Agilent 5975C MS with source and quadrupole mass filter temperature setting of 230°C and 150°C, respectively. Mass spectra were collected in scanning ion mode (*m/z* 50 and 500) in ChemStation (Agilent) and deconvoluted using AnalyzerPro (SpectralWorks, Runcorn, UK). Putative peak identities were assigned based on the NIST08, Fiehnlib (Agilent) and in-house libraries from authentic standards using AnalyzerPro. Before and after each suite of sample injections, a mix containing sucrose, fructose, glutamic acid, succinic acid and ascorbic acid was run for monitoring derivatization, instrument performance and retention time shifts. All processed metabolite data are provided in **Supplementary Dataset 1**.

Metabolite concentrations were calculated on a bulk leaf water content basis following the conversion of peak areas to metabolite mass contents using standard curves constructed from authentic standards. Bulk organic solute concentration data for comparative purposes were obtained using a suite of 10 metabolites which comprised 93–95% of total chromatogram peak area. The metabolites used comprised only those of well-known retention time and library match confidences from previous work in our lab (Jeong et al. 2004, Frost et al. 2012, Xue et al. 2013, Bowsher et al. 2015). Purified salicortin was the kind gift of Richard L. Lindroth (University of Wisconsin).

### Petiole exudates and sap

Three days in advance of excision for petiole exudate collection, lamina of fully expanded source leaves were trimmed to a 3 × 8 cm triangle along the midvein. Following mid-afternoon excision from the plant, petiole ends were trimmed under 5 mM EDTA ethylenediaminetetraacetic acid (EDTA) and allowed to soak for 1 h. Petiole ends were then dipped twice in deionized water, tapped to remove excess water and submerged in microcentrifuge tubes containing 250 µl of 500 μM EDTA. To minimize uptake, exudate collection was carried out in the dark in a humid chamber. After ∼4 h collection period, petioles were removed and freeze-dried for subsequent GC-MS analysis of tissue extracts. Exudate solution was dried in a centrivap, resuspended in 100 μl of 50% methanol/H_2_O containing loading standard adonitol, re-dried and derivatized for GC-MS as above. Separately, petiole saps for subsequent metabolite (sucrose) comparisons with stem xylem saps were collected using a pressure bomb (PMS Instruments, Albany, OR, 97322, USA). Fully expanded leaves were sliced off near the base of the petiole with a razor blade, the shaft of the petiole was wiped with a damp cloth, the lamina end was inserted into the pressure device chamber and a pressure of ∼0.8 MPa was applied until the exudate appeared at the cut surface. A kimwipe was used to remove the initial several microliters, after which a pipette was used to collect about 10–20 μl for metabolic analysis.

### Stem xylem sap

Saplings about 1.5 m in height with a basal stem diameter of ∼1.5 cm were smoothly coppiced about 15 cm above the soil surface using a razor blade. Bark was removed from the top 1.5 cm of the stump, all surfaces of the protruding wood were tamped clean with a damp cloth and xylem sap was allowed to collect at the top of the stump. A kimwipe was used to remove the first 0.2–0.5 ml of sap, after which a pipette was used to collect 100–300 μl of sap as it emerged over the next 15–20 min. Sap was snap frozen for metabolic analysis.

### Condensed tannins

Tissue-bound CTs were analyzed by extracting 10 mg freeze-dried tissue powder in 600 μl of methanol for 15 min in an ultrasonic bath and centrifuging at 15,000 g for 10 min to remove pigments (Harding et al. 2005). The depigmented pellet was dried for CT analysis using the butanol–HCl method (Porter et al. 1986). Hydrolysis was carried out at 95°C for 20 min in 1 ml butanol–5% hydrochloric acid containing ferric ammonium sulfate, and absorbance (A_550_) was read and quantified against aspen leaf CT standards.

### Hydrogen peroxide

Hydrogen peroxide (H_2_O_2_) contents were measured using the Amplex Red™ Hydrogen Peroxide Assay Kit (Invitrogen, Carlsbad, CA, 92008, USA), following manufacturer’s instructions.

### Chlorophyll fluorescence

Chlorophyll fluorescence transients were obtained using a hand-held FluorPen, FP-100 (Qubit Instruments, Kingston, ON, Canada) as previously described (Harding et al. 2009).

### Statistics

One-way analysis of variance (ANOVA) with Holm–Sidak post hoc testing (SigmaStat 4.0, Systat Software, San Jose, CA, 95110, USA) was used for multi-group comparisons at each sampling date indicated in **Fig. 1** with genotype pooling as indicated in respective figures. Means and standard deviations for unpooled data are based on *n* = 4 independent biological replicates for each genotype (line). Regression slope analysis was done using Excel Analysis Tool Pak. Significance testing for difference between slopes was carried out using https://www.danielsoper.com/statcalc/calculator.aspx.

## Supplementary Material

pcac087_Supp

## Data Availability

Source data from GC-MS metabolite profiling are provided in **Supplementary Dataset 1**.
