# Supplementary material for: Tonoplast Sucrose Trafficking Modulates Starch Utilization and Water Deficit Behavior in Poplar Leaves
Source: Plant Cell Physiol. 2022 Jun 21;63(8):1117–29. doi: 10.1093/pcp/pcac087 (PMC9381566; doi:10.1093/pcp/pcac087)
Supplement: pcac087_Supp [file pcac087_supp.zip › pcac087_Supp/pcp-2022-e-00123-File011.pdf]

A

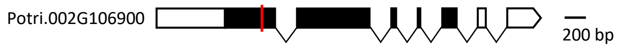

B

Line	gRNA: GTCATTCCGCTGACATT.GGGTGG	Read #	Indel/WT %	Pattern
WT	CGGTTTTGATTATCGGTCATTCCGCTGACATT.GGGTGGTGGTTAGGCGATAGAGGAGGTGTTAGACCAA	1722	99.4	WT
	CGGTTTTGATTATCGGTCATTCCGCTGACATT.GGGTGGTGGTTAGGCGATAGAGGAGGTGTTAGGCCAA	1736	98.7	WT
Cas9	CGGTTTTGATTATCGGTCATTCCGCTGACATT.GGGTGGTGGTTAGGCGATAGAGGAGGTGTTAGACCAA	1123	99.3	WT
	CGGTTTTGATTATCGGTCATTCCGCTGACATT.GGGTGGTGGTTAGGCGATAGAGGAGGTGTTAGGCCAA	1132	98.5	WT
**KO-1**	CGGTTTTGATTATCGGTCATTCCGCTGACATTTGGGTGGTGGTTAGGCGATAGAGGAGGTGTTAGACCAA	1355	99.5	+1
	CGGTTTTGATTATCGGTCATTCCGCTGACAT-.GGGTGGTGGTTAGGCGATAGAGGAGGTGTTAGGCCAA	1452	98.7	-1
KO-5	CGGTTTTGATTATCGGTCATTCCGCTGACATTTGGGTGGTGGTTAGGCGATAGAGGAGGTGTTAGACCAA	1036	99.4	+1
	CGGTTTTGATTATCGGTCATTCCGCTGACAT-.GGGTGGTGGTTAGGCGATAGAGGAGGTGTTAGGCCAA	1083	98.9	-1
KO-6	CGGTTTTGATTATCGGTCATTCCGCTGACA--.GGGTGGTGGTTAGGCGATAGAGGAGGTGTTAGACCAA	1041	99.3	-2
	CGGTTTTGATTATCGGTCATTCCGCTGACATTTGGGTGGTGGTTAGGCGATAGAGGAGGTGTTAGGCCAA	983	99.2	+1
**KO-7**	CGGTTTTGATTATCGGTCATTCCGCTGACATTTGGGTGGTGGTTAGGCGATAGAGGAGGTGTTAGACCAA	1057	99.6	+1
	CGGTTTTGATTATCGGTCATTCCGCTGACAT-.GGGTGGTGGTTAGGCGATAGAGGAGGTGTTAGGCCAA	1173	99.3	-1
KO-8	CGGTTTTGATTATCGGTCATTCCGCTGACAT-.GGGTGGTGGTTAGGCGATAGAGGAGGTGTTAGACCAA	1127	99.4	-1
	CGGTTTTGATTATCGGTCATTCCGCTGACATTTGGGTGGTGGTTAGGCGATAGAGGAGGTGTTAGGCCAA	1137	99.0	+1
KO-10	CGGTTTTGATTATCGGTCATTCCGCTGAC---.GGGTGGTGGTTAGGCGATAGAGGAGGTGTTAGACCAA	1526	99.3	-3
	CGGTTTTGATTATCGGTCATTCCGCTGACATTTGGGTGGTGGTTAGGCGATAGAGGAGGTGTTAGGCCAA	1569	99.6	+1
KO-11	CGGTTTTGATTATCGGTCATTCCGCTGACATTTGGGTGGTGGTTAGGCGATAGAGGAGGTGTTAGACCAA	1498	99.4	+1
	CGGTTTTGATTATCGGTCATTCCGCTGACATT.GG------------CGATAGAGGAGGTGTTAGGCCAA	1580	99.2	-12
KO-12	CGGTTTTGATTATCGGTCATTCCGCTGAC---.GGGTGGTGGTTAGGCGATAGAGGAGGTGTTAGACCAA	1683	99.3	-3
	CGGTTTTGATTATCGGTCATTCCGCTGACAT-.GGGTGGTGGTTAGGCGATAGAGGAGGTGTTAGGCCAA	1566	99.6	-1
KO-14	CGGTTTTGATTATCGGTCATTCCGCTGAC---.GGGTGGTGGTTAGGCGATAGAGGAGGTGTTAGACCAA	1077	99.4	-3
	CGGTTTTGATTATCGGTCATTCCGCTGA----.-GGTGGTGGTTAGGCGATAGAGGAGGTGTTAGGCCAA	1195	98.8	-5
KO-18	CGGTTTTGATTATCGGTCATTCCGCTGACATTTGGGTGGTGGTTAGGCGATAGAGGAGGTGTTAGACCAA	1163	99.7	+1
	CGGTTTTGATTATCGGTCATTCCGCTGACATTTGGGTGGTGGTTAGGCGATAGAGGAGGTGTTAGGCCAA	1313	99.5	+1
KO-19	CGGTTTTGATTATCGGTCATTCCGCTGA---T.GGGTGGTGGTTAGGCGATAGAGGAGGTGTTAGACCAA	787	99.4	-3
	CGGTTTTGATTATCGGTCATTCCGCTGACATTTGGGTGGTGGTTAGGCGATAGAGGAGGTGTTAGGCCAA	730	99.9	+1
KO-21	CGGTTTTGATTATCGGTCATTCCGCTGA---T.GGGTGGTGGTTAGGCGATAGAGGAGGTGTTAGACCAA	1957	99.5	-3
	CGGTTTTGATTATCGGTCATTCCGCTGACATTTGGGTGGTGGTTAGGCGATAGAGGAGGTGTTAGGCCAA	1770	99.5	+1
KO-25	CGTTTTGATTATCGGTCATTCCGCTGACAATT.GGCGCAATATTGTGTTTGGGTTTGGGTTAGGCGATAG……ACCAA	1617	98.5	-8, +25
	CGGTTTTGATTATCGGTCATTCCGCTGACAT-.GGGTGGTGGTTAGGCGATAGAGGAGGTGTTAGGCCAA	1989	99.5	-1
KO-32	CGGTTTTGATTATCGGTCATTCCGCTGACA--.GGGTGGTGGTTAGGCGATAGAGGAGGTGTTAGACCAA	1623	99.4	-2
	CGGTTTTGATTATCGGTCATTCCGCTGA---T.GGGTGGTGGTTAGGCGATAGAGGAGGTGTTAGGCCAA	1678	99.4	-3
KO-33	CGGTTTTGATTATCGGTCATTCCG--------.-----GTGGTTAGGCGATAGAGGAGGTGTTAGACCAA	2813	99.8	-13
	CGGTTTTGATTATCGGTCATTCCGCTGACATTTGGGTGGTGGTTAGGCGATAGAGGAGGTGTTAGGCCAA	2514	99.4	+1
KO-35	CGGTTTTGATTATCGGTCATTCCGCTGACATTTGGGTGGTGGTTAGGCGATAGAGGAGGTGTTAGACCAA	2093	99.4	+1
	CGGTTTTGATTATCGGTCATTCCGCTGACAT-.GGGTGGTGGTTAGGCGATAGAGGAGGTGTTAGGCCAA	2163	99.6	-1
KO-36	CGGTTTTGATTATCGGTCATTCCGCTGACATTTGGGTGGTGGTTAGGCGATAGAGGAGGTGTTAGACCAA	2103	99.7	+1
	CGGTTTTGATTATCGGTCATTCCGCTGACAT-.GGGTGGTGGTTAGGCGATAGAGGAGGTGTTAGGCCAA	2173	99.6	-1
KO-37	CGGTTTTGATTATCGGTCATTCCGCTGAC--T.GGGTGGTGGTTAGGCGATAGAGGAGGTGTTAGACCAA	1993	99.2	-2
	CGGTTTTGATTATCGGTC-------------T.GGGTGGTGGTTAGGCGATAGAGGAGGTGTTAGGCCAA	2061	99.4	-13
KO-38	CGGTTTTGATTATCGGTCATTCCGCTGAC--T.GGGTGGTGGTTAGGCGATAGAGGAGGTGTTAGACCAA	857	99.6	-2
	CGGTTTTGATTATCGGTCATTCCGCTGACATTTGGGTGGTGGTTAGGCGATAGAGGAGGTGTTAGGCCAA	784	99.6	+1
KO-40	CGGTTTTGATTATCGGTCATTCCGCTGACATTTGGGTGGTGGTTAGGCGATAGAGGAGGTGTTAGACCAA	1626	99.7	+1
	CGGTTTTGATTATCGGTCATTCCGCTGACATTTGGGTGGTGGTTAGGCGATAGAGGAGGTGTTAGGCCAA	1615	99.3	+1
KO-44	CGGTTTTGATTATCGGTCATTCCGCTGACATT.--GTGGTGGTTAGGCGATAGAGGAGGTGTTAGACCAA	1642	99.6	-2
	CGGTTTTGATTATCGGTCATTCCGCTGACAT-.GGGTGGTGGTTAGGCGATAGAGGAGGTGTTAGGCCAA	1554	99.4	-1
KO-45	CGGTTTTGATTATCGGTCATTCCGCTGACATTTGGGTGGTGGTTAGGCGATAGAGGAGGTGTTAGACCAA	1641	99.5	+1
	CGGTTTTGATTATCGGTCATTCCGCTGA----.GGGTGGTGGTTAGGCGATAGAGGAGGTGTTAGGCCAA	1583	99.4	-4
KO-47	CGGTTTTGATTATCGGTCATTCCGCTGACATTTGGGTGGTGGTTAGGCGATAGAGGAGGTGTTAGACCAA	949	99.6	+1
	CGGTTTTGATTATCGGTCATTCCGCTGACATTTGGGTGGTGGTTAGGCGATAGAGGAGGTGTTAGGCCAA	946	99.5	+1
KO-49	CGGTTTTGATTATCGGTCATTCCGCTGACATT.-GGTGGTGGTTAGGCGATAGAGGAGGTGTTAGACCAA	1097	99.4	-1
	CGGTTTTGATTATCGGTCATTCCGCTGACAT-.GGGTGGTGGTTAGGCGATAGAGGAGGTGTTAGGCCAA	1117	99.6	-1
KO-50	CGGTTTTGATTATCGGTCATTCCGCTGAC--T.GGGTGGTGGTTAGGCGATAGAGGAGGTGTTAGACCAA	920	99.6	-2
	CGGTTTTGATTATCGGTCATTCCGCTGACATTTGGGTGGTGGTTAGGCGATAGAGGAGGTGTTAGGCCAA	867	99.5	+1
KO-51	CGGTTTTGATTATCGGTCATTCCGCTGACATTTGGGTGGTGGTTAGGCGATAGAGGAGGTGTTAGACCAA	1202	99.9	+1
	CGGTTTTGATTATCGGTCATTCCGCTGACATTTGGGTGGTGGTTAGGCGATAGAGGAGGTGTTAGGCCAA	1252	99.8	+1
KO-52	CGGTTTTGATTATCGGTCATTCCGCTGACATTTGGGTGGTGGTTAGGCGATAGAGGAGGTGTTAGACCAA	1028	99.1	+1
	CGGTTTTGATTATCGGTCATTCCGCTGACATTGGGGTGGTGGTTAGGCGATAGAGGAGGTGTTAGGCCAA	964	99.1	+1
KO-55	CGGTTTTGATTATCGGTCATTCCGCTGAC--T.GGGTGGTGGTTAGGCGATAGAGGAGGTGTTAGACCAA	805	99.1	-2
	CGGTTTTGATTATCGGTCATTCCGCTGACATTTGGGTGGTGGTTAGGCGATAGAGGAGGTGTTAGGCCAA	876	99.8	+1
KO-57	CGGTTTTGATTATCGGTCATTCCGCTGACATTTGGGTGGTGGTTAGGCGATAGAGGAGGTGTTAGACCAA	926	99.2	+1
	CGGTTTTGATTATCGGTCATTCCGCTGACATTAGGGTGGTGGTTAGGCGATAGAGGAGGTGTTAGGCCAA	1011	99.6	+1
KO-60	CGGTTTTGATTATCGGTCATTCCGCTGACAT-.GGGTGGTGGTTAGGCGATAGAGGAGGTGTTAGACCAA	1951	99.4	-1
	CGGTTTTGATTATCGGTCATTCCGCTGACATTTGGGTGGTGGTTAGGCGATAGAGGAGGTGTTAGGCCAA	1915	99.4	+1

C

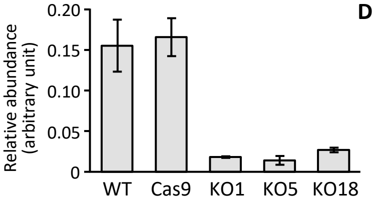

D

```
WT  ATTATCGGTCATTCCGCTGACATT.GGGTGGTGGTTAGGCGATAGAGGAGGTGT
     I  I  G  H  S  A  D  I   G  W  W  L  G  D  R  G  G  V
+1  ATTATCGGTCATTCCGCTGACATTTGGGTGGTGGTTAGGCGATAGAGGAGGTGT
     I  I  G  H  S  A  D  I  W  V  V  V  R  R  *
-1  ATTATCGGTCATTCCGCTGACAT-.GGGTGGTGGTAGGCGATAGAGGAGGTGT
     I  I  G  H  S  A  D  M    G  G  G  *
-2  ATTATCGGTCATTCCGCTGACA--.GGGTGGTGGTTAGGCGATAGAGGAGGTGT
     I  I  G  H  S  A  D       R  V  V  V  R  R  *
```

Fig. S1. Characterization of *SUT4*-KO mutants. **A**, *PtaSUT4* gene structure. Exons are shown in boxes (noncoding regions in white) and the gRNA target site in red (introns not drawn to scale). **B**, Editing patterns determined by amplicon sequencing. Lines used in the greenhouse experiments are boldface. The gRNA site is shown on top (PAM underlined) and mutations in red. **C**, *PtaSUT4* transcript levels in WT, Cas9 and three representative KO lines. Values represent mean ± SE of *n* = 5-6 control or *n* = 3-4 KO plants. **D**, Theoretical translation of WT and the top three mutant alleles. gRNA target site is shaded in grey and Indels and stop codons are shaded in red.

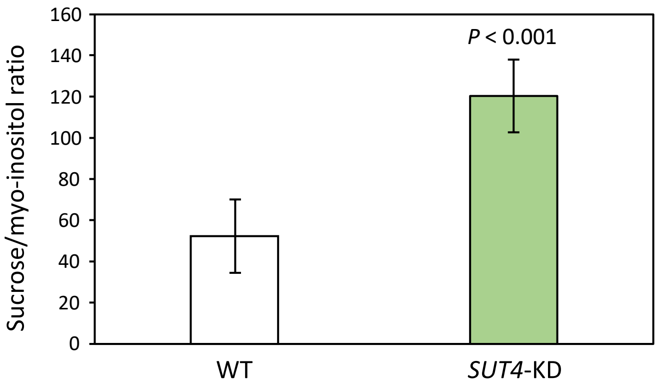

Fig. S2. Sucrose enrichment in petiole exudates of WT and *SUT4*-KD line G leaves. Enrichment is expressed as peak area ratio of sucrose/myo-inositol. Myo-inositol and sucrose peak areas were obtained from GC-MS metabolic profiles of the exudates. Myo-inositol peak areas were nearly identical for both lines. Histogram bars represent the mean±SD of n=6 WT leaves and 4 KD leaves. Leaves were excised for exudate collection during the afternoon. Significance was determined by the two-sample *t*-test.

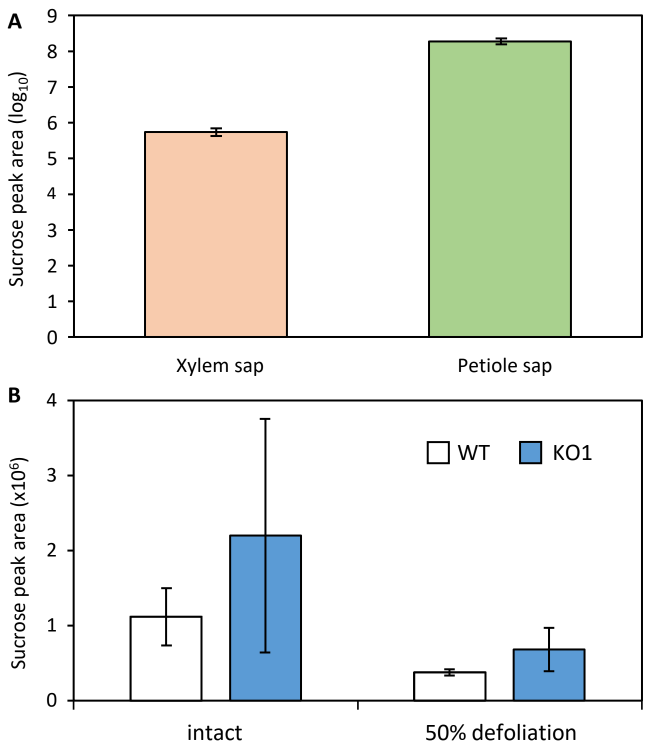

Fig. S3. Relative sucrose abundance of petiole and stem xylem saps. **A**, Petiole versus xylem sap sucrose. Values represent mean±SD of n=9 WT petiole sap samples collected at dawn using a pressure bomb and n=9 WT stem xylem sap samples from the same plants collected shortly after dawn. **B**, Relative sucrose abundance in xylem sap of WT, KD and KO1. Histogram bars represent mean±SD of n=3 plants for each genotype x defoliation level combination. Plants were partially defoliated by removing every other fully expanded leaf two weeks prior to xylem sap collection.

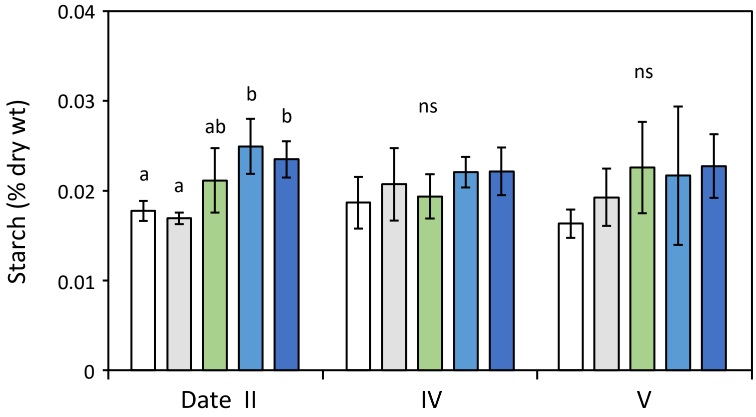

Fig. S4. Midvein starch content of all lines at dates II, IV, and V. Skies were clear for these three dates, but soil water content was approximately 15% and 30% lower on dates IV and V, respectively, than date II. Histogram bars and significance testing as in Fig. 2.

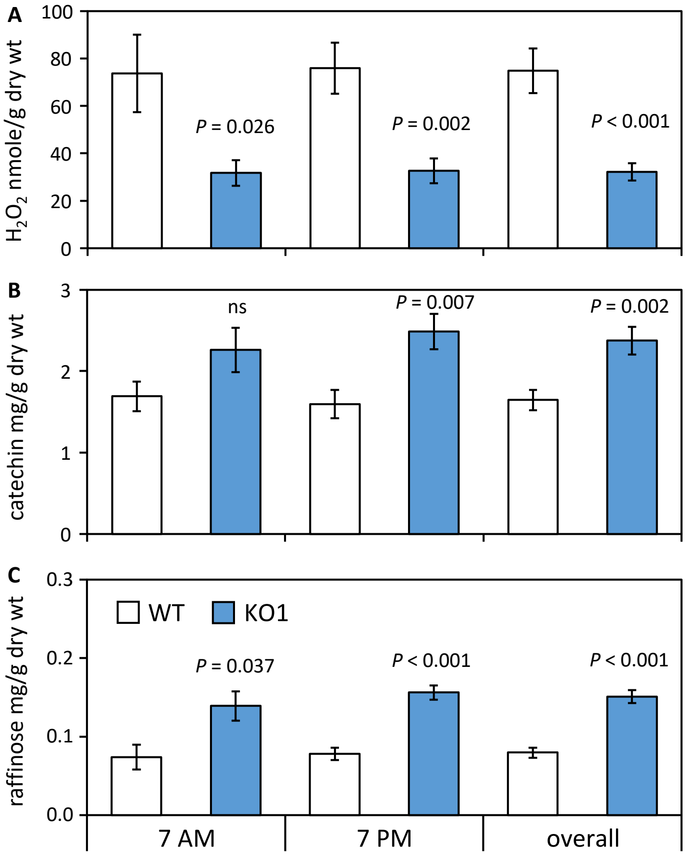

Fig. S5. Hydrogen peroxide levels correlated negatively with antioxidant levels and were higher in WT than KO source leaves used in benchtop drying experiments. **A**, Hydrogen peroxide content. **B**, Catechin content. **C**, Raffinose content of source leaves excised at 7 AM or 7 PM. Each histogram bar represents the mean±SE of n=9. Significance was determined by the two-sample *t*-test for leaves excised at 7 AM, 7 PM or both times pooled (overall).

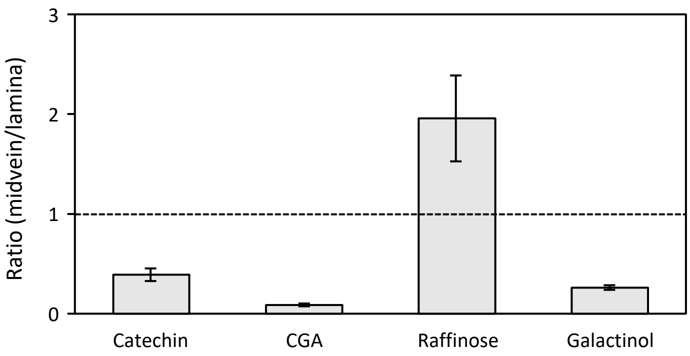

Fig. S6. Midvein antioxidant abundance as a fraction of lamina abundance. Freeze-dried tissue powders were profiled by GC-MS for the analysis. Histogram bars represent mean±SEM of n=13 ratios from 4 WT-Cas9, 13 KD and 6 KO plants. Dashed line represents an midvein/lamina peak area ratio of 1.0.

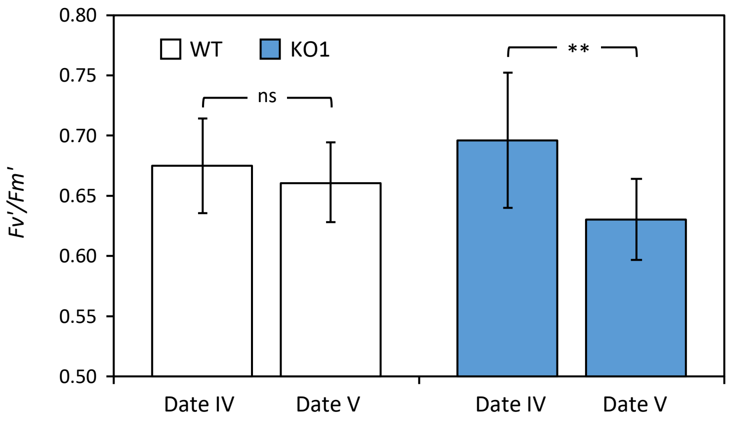

Fig. S7. Chlorophyll fluorescence at dates IV and V. Photosystem II quantum yield was estimated as *Fv'/Fm'* where *Fv'* is chlorophyll variable fluorescence in the light-adapted state and *Fm'* is the maximum fluorescence yield in the light-adapted state. Histogram data represent the mean±SD of n= 8-10 determinations. For each determination, four readings were collected from each leaf and averaged. Significance was determined for the difference between lines (none was significant at each date) or between dates for each genotype using the two-sample *t*-test (**, $P < 0.01$).
